# Supplementary material for: Cardiogenic programming of human pluripotent stem cells by dose-controlled activation of EOMES
Source: Nat Commun. 2018 Jan 30;9:440. doi: 10.1038/s41467-017-02812-6 (PMC5789885; doi:10.1038/s41467-017-02812-6)
Supplement: Supplementary file 1 — Supplementary Information [file 41467_2017_2812_MOESM1_ESM.pdf]

Cardiogenic programming of human pluripotent stem cells by a single transcription factor

Martin J. Pfeiffer, Roberto Quaranta, Ilaria Piccini, Jakob Fell, Jyoti Rao, Albrecht Röpke, Guiscard Seebohm and Boris Greber

SUPPLEMENTARY INFORMATION

- Supplementary Figures

- Supplementary Tables

Supplementary Figure 1 Differentiation of WT and EOMES KO hESCs under conditions permissive for neural or non-cardiac mesodermal fates.

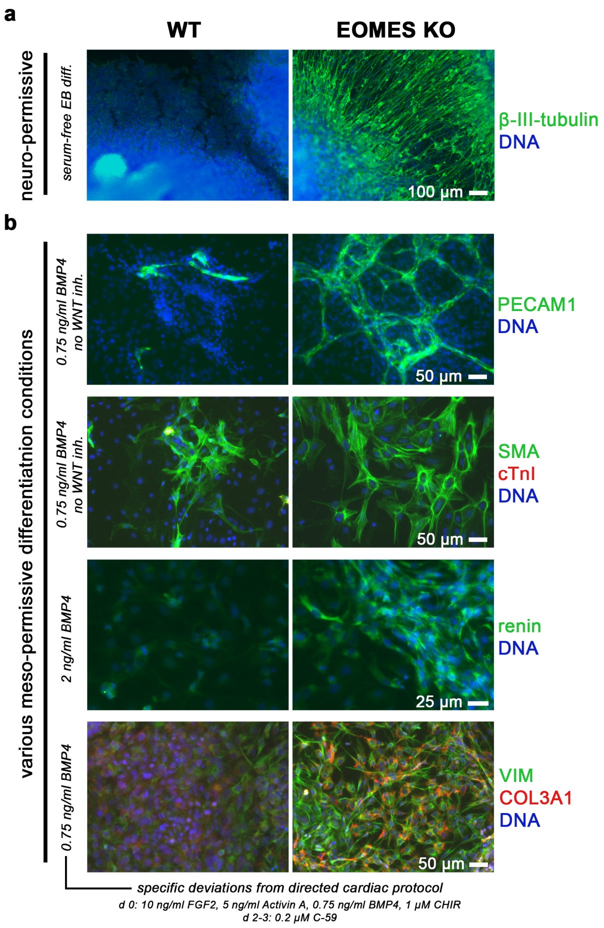

(a) Using spontaneous differentiation conditions, EOMES KO cells displayed accelerated differentiation into neurons at 2 wk as compared to WT controls. **(b)** EOMES KO cells are overall competent for differentiating into several non-cardiac mesoderm derivatives. SMA = smooth muscle actin, cTnI = cardiac troponin I, VIM = vimentin, COL3A1 = type III α 1 collagen. Specific information on differentiation conditions is given on the left.

Supplementary Figure 2 Optimised generation and specific characterisation of DOX-induced cardiomyocytes.

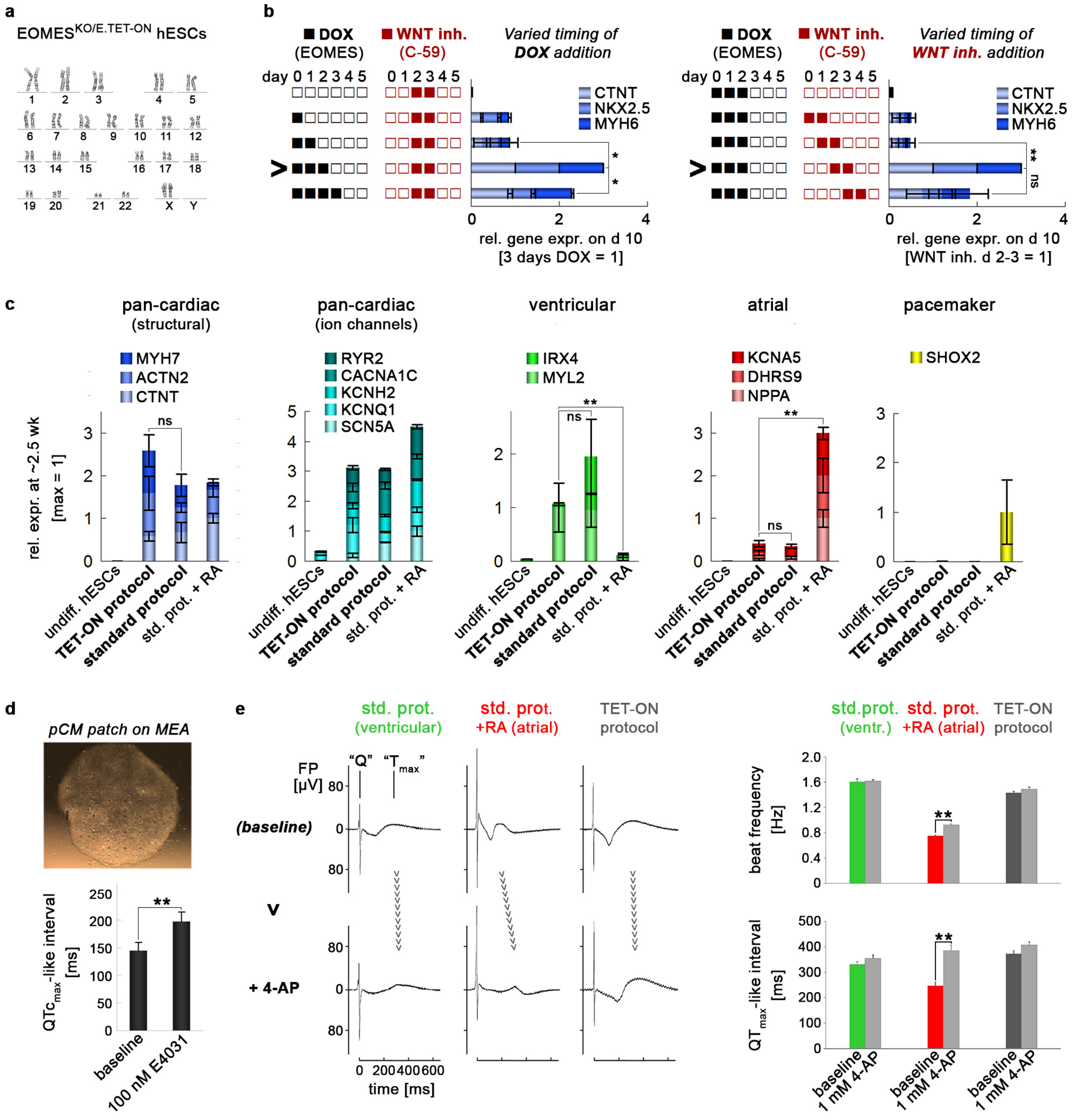

(a) Normal female karyotype (n = 10) of clonal EOMES$^{KO/E.TET-ON}$ hESCs (HuES6 background). **(b)** Optimisation of parameters in the TET-ON protocol. Left: Variation of time of exposure to doxycycline (qPCR data, n = 3-5 per condition). Right: Sliding window experiment to optimise the timing of the WNT signaling inhibition step (n = 2-5). Arrow heads indicate the best conditions subsequently used throughout. **(c)** Cardiac marker expression comparison between CMs generated with the TET-ON protocol, or with the standard growth factor-based protocol, or using additional retinoic acid (RA) supplementation to promote an atrial CM fate (qPCR data, all HuES6 background, n = 2-4 per data point). **(d)** Multielectrode array-based validation of hERG potassium channel functionality in pCMs. Top: Electrode chip with patch of beating pCMs. Bottom: hERG channel blocker E-4031 (100 nM) significantly prolongs field potential durations quantified as frequency-corrected QT_{max}-like intervals (n = 4). **(e)** Functional investigation of pCM subtype identity based on 4-aminopyridine-mediated inhibition of the atrial-specific KCNA5 channel. 4-AP treatment shifts the indicated T wave-like signal to a later time-point in atrial-like hESC-CMs but virtually not in standard hESC-CMs or pCMs (n = 8, 11, and 14, respectively. Comparatively slow spontaneous beating, together with resistance to 4-AP, and considering the data of panel b / Fig. 2c collectively indicates an overall ventricular-like default phenotype of pCMs. All error bars in this figure: s.e.m..

Supplementary Figure 3 EOMES expression level dependency of distinct differentiation fates in independent cell lines.

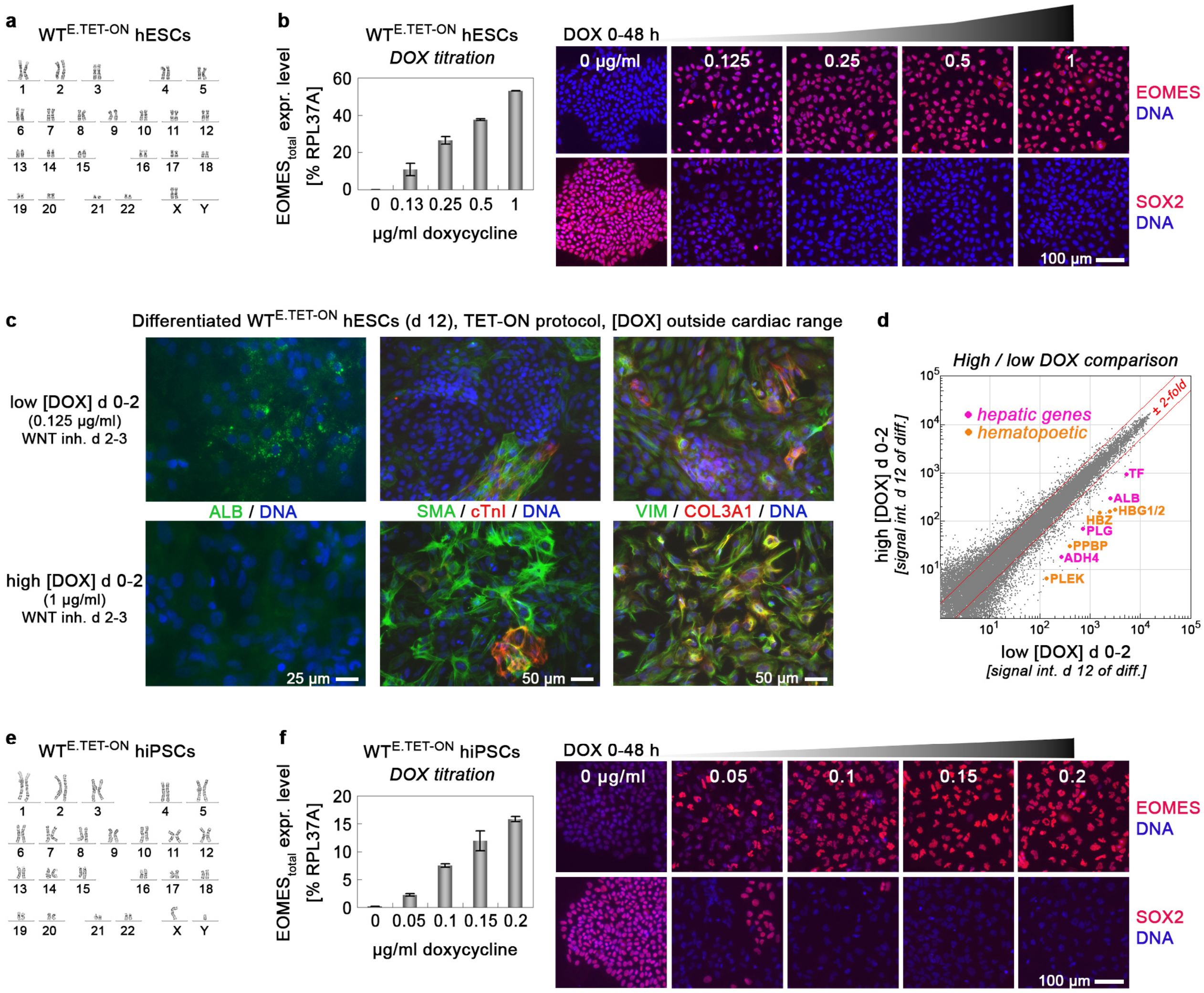

(a) Normal female karyotype (n = 10) of clonal WT$^{E.TET\text{-}ON}$ hESCs (HuES6 background). **(b)** DOX titration of these WT$^{E.TET\text{-}ON}$ hESCs validates dose-dependent transgene induction and concomitant SOX2 repression as a key downstream effect (analysed at 48 h, n = 2; error bars: s.e.m.). **(c)** Carrying out the TET-ON protocol with DOX concentrations below or above the cardiac range promotes distinct endodermal and non-cardiac mesoderm fates. ALB = albumin, SMA = smooth muscle actin, cTnI = cardiac troponin I, VIM = vimentin, COL3A1 = type III α 1 collagen. **(d)** Microarray-based comparison of samples treated as in panel c. Low-DOX conditions appear to promote hematopoetic differentiation in addition to hepatic outcomes. **(e)** Normal male karyotype of clonal WT$^{E.TET\text{-}ON}$ hiPSCs (n = 10). **(f)** DOX titration of these WT$^{E.TET\text{-}ON}$ hiPSCs validates dose-dependent transgene induction and concomitant SOX2 repression as a key downstream effect (analysed at 48 h, n = 2; error bars: s.e.m.). Note the persistent expression of SOX2 at 0.05 μg/ml DOX due to heterogeneous EOMES induction at this borderline concentration.

Supplementary Figure 4 Implication of WNT signaling in EOMES-driven cardiac induction.

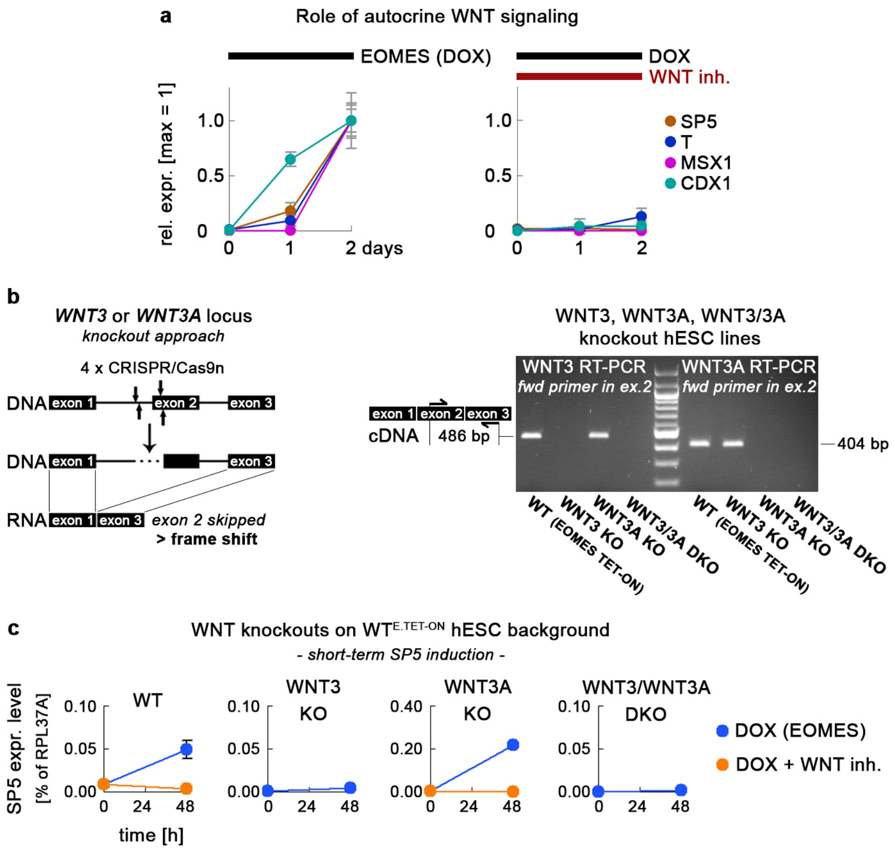

(a) DOX-mediated EOMES expression induces known WNT target genes within 2 days (left) and this effect is suppressed in the presence of a WNT inhibitor (C-59) that antagonises the secretion of WNT ligands (right; n = 3, normalised to no-WNT-inh. d 2; error bars: s.e.m.). **(b)** Left: Schematic of CRISPR-mediated disruption strategy of the *WNT3* and *WNT3A* genes. Exon / intron sizes are not drawn to scale. See Supplementary Table 1 for deleted genomic sequences. Right: Diagnostic RT-PCRs on WT and clonal knockout cell lines using exon 2-specific forward primers that failed to amplify in the correct KO samples. **(c)** Early DOX-mediated induction of *SP5* is dependent on the ligand-encoding *WNT3* gene but not on *WNT3A*, because of the lack of induction in WNT3 knockout and WNT3/WNT3A double-knockout cells under DOX (qPCR data, n = 2; error bars: s.e.m.). WNT inhibitor-treated samples reconfirm *SP5* as a WNT target gene.

Supplementary Figure 5 Uncropped immunoblots.

> Fig. 1a

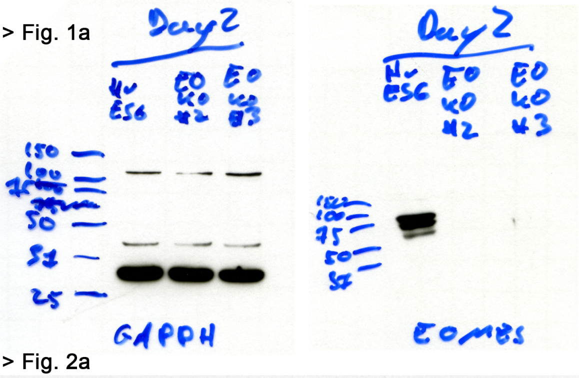

> Fig. 2a

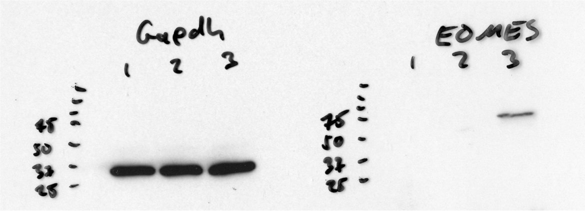

> Fig. 2b

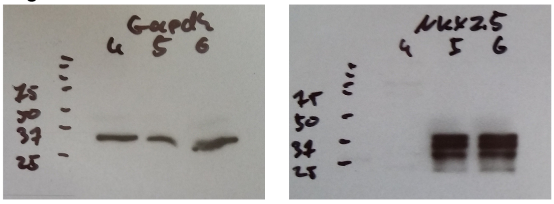

Supplementary Table 1 Genomic DNA sequences excised using CRISPR/Cas9.

Knockout cell line	Deleted genomic sequence in FIRST *WNT3* allele	Deleted genomic sequence in SECOND *WNT3* allele
WNT3 KO	AGGGACCTGCAGGCAGACAGAGGGTAGTAACACTGTGGGCAC<br>AAAGCACAGAGCCCATCCTGGGCACCATGGCCGCTTTGTGAA<br>CCCTCCGGGGTAGGTGGAGAGGCAGAGGGCC	AGGGACCTGCAGGCAGACAGAGGGTAGTAACACTGTGGGCAC<br>AAAGCACAGAGCCCATCCTGGGCACCATGGCCGCTTTGTGAA<br>CCCTCCGGGGTAGGTGGAGAGGCAGAGGGCC
WNT3A KO	N/A	N/A
WNT3 / WNT3A DKO	TGAGCCCAGAGATGTGTACTGCTGGCCCAGGGCCAGGGACCT<br>GCAGGCAGACAGAGGGTAGTAACACTGTGGGCACAAAGCACA<br>GAGCCCATCCTGGGCACCATGGCCGCTTTGTGAACCCTCCGG<br>GGTAGGTGGAG	GCTGTGAGCCCAGAGATGTGTACTGCTGGCCCAGGGCCAGGG<br>ACCTGCAGGCAGACAGAGGGTAGTAACACTGTGGGCACAAAG<br>CACAGAGCCCATCCTGGGCACCATGG

Knockout cell line	Deleted genomic sequence in FIRST *WNT3A* allele	Deleted genomic sequence in SECOND *WNT3A* allele
WNT3 KO	N/A	N/A
WNT3A KO	TAGCCTGCTCATCTGTGTCGCTGTCCCAGCCCCACACTCACCA<br>CCGGATCTTGCCCTCTGCAGGTCGCTGGCTGTTGGGCCACAGT<br>ATTCCTCCCTGGGCTCGCAGCCCATCCTGTGTGCCAGCATCCC<br>GGGCCTGGTCCCCAAGCAGCTCCGCTTCTG	TAGCCTGCTCATCTGTGTCGCTGTCCCAGCCCCACACTCACCA<br>CCGGATCTTGCCCTCTGCAGGTCGCTGGCTGTTGGGCCACAGT<br>ATTCCTCCCTGGGCTCGCAGCCCATCCTGTGTGCCAGCATCCC<br>GGGCCTGGTCCCCAAGCAGCTCCGCTTCTG
WNT3 / WNT3A DKO	AGCAAAGGGTCTGTAGCCTGCTCATCTGTGTCGCTGTCCCAGC<br>CCCACACTCACCACCGGATCTTGCCCTCTGCAGGTCGCTGGC<br>TGTTGGGCCACAGTATTCCTCCCTGGGCTCGCAGCCCATCCTG<br>TGTGCCAG	AGCAAAGGGTCTGTAGCCTGCTCATCTGTGTCGCTGTCCCAGC<br>CCCACACTCACCACCGGATCTTGCCCTCTGCAGGTCGCTGGC<br>TGTTGGGCCACAGTATTCCTCCCTGGGCTCGCAGCCCATCCTG<br>TGTGCCAG

Supplementary Table 2 Oligonucleotides used in this study.

Gene	Product size (bp)	Fwd primer	Rev primer	Remarks
**Primers for RT-qPCR**				
ACTN2	75	GCCAGAGAGAAGGATGCAATCAC	AAGCATGGGAACCTGGAATCAA	
CACNA1C	119	CCAGGCTCCACGACTTCACA	GGCCTTTCTCGAGGGTGAGA	
CDX1	86	TGAGGAGGGAGGAACGTGGT	GGGCTCAGTGCCCTTATGATG	
CTNT	87	GGCAGCTCCTGTTTGGAAATG	TTATTACTGGTGTGGAGTGGGTGTG	
DHRS9	119	TCCGGTGGTAGAGTGCATGG	GCAAAGCTGCTGGCATGTG	
EOMES CDS	76	CGGCCTCTGTGGCTCAAA	AAGGAAACATGCGCCTGC	total EOMES
EOMES UTR	71	CTTGCTAGGCCTCTGCTGTGTG	TTGGTGACTCCTTAGCTTGCTCTCT	endogenous-specific
IRX4	77	CGGAGCAGAAGAGGCCAGAT	CAGAACGGAACCGCCTTCTC	
KCNA5	86	CCAGCAGAGGGATAACCCAAAC	TTGGAACACATGGATGGAGGAG	
KCNH2	115	CGGTGCATGTGTGGTCTTGA	TGACATCTGCCTGCACCTGA	
KCNQ1	113	GCAGCCAGCCAAACACACA	GCGATGTAATGCCCAGAAGGA	
MSX1	71	AACCCTCACACTGCTCCAGTTTC	TTTGGCAGGGATCAGACTTCG	
MYH6	101	ACCTGGTGGACAAGCTGCAA	CACCTTGCGGAACTTGGACA	
MYH7	74	TTGATCTGCTCAGCCCTGGA	GCTTCCTCCCAAGGAGCTGTT	
MYL2 / MLC2v	73	TGGTCCCTGCCCTCATCTCT	GGCAGCCACATGGCTAACAG	
MYL3	87	AAGTTGATGGCTGGGCAAGA	GCACGAGGTTTAGCTGGACA	
MYL4	87	CTGGGCAAGAGGATGCCAAT	GCACCTGGAAGACTCTGCTTCA	
NKX2-5	96	ACCGATCCCACCTCAACAGC	CTCCGCAGGAGTGAATGCAA	
NPPA / ANP	77	GCTGCAGCTTCCTGTCAACACT	AGGCGAGGAAGTCACCATCAA	
RPL37A	84	GTGGTTCCTGCATGAAGACAGTG	TTCTGATGGCGGACTTTACCG	housekeeping gene used for normalization
RYR2	83	GGAGCCAGTGTCATCCACCA	CAGGTGGCTGAAAGAATGAGCA	
SCN5A	77	ACTGCACAATGACCAGCAGGA	GTGAGAAGTGCTCGATTAGTTCAGACA	
SHOX2	119	TCTGTATCCCAATTCGCTAGCAA	CAACCGCATACCAAAGTTCAGTT	
SP5	104	TTCCAACTTCCGCTGCCTTC	CGTACGGCAGAGCTCCCAAT	
T	79	CCTTGCTCACACCTGCAGTAGC	GGCCAACTGCATCATCTCCA	
**Oligonucleotides for CRISPR gRNAs**				
WNT3 1/1'	n/a	GGCTCACAGCCCCTGCTCTG	GAGATGTGTACTGCTGGCCC	first WNT3 CRISPR/Cas9n pair
WNT3 2/2'	n/a	GAGGGTTCACAAAGCGGCCA	GGGGTAGGTGGAGAGGCAGA	second WNT3 CRISPR/Cas9n pair
WNT3A 1/1'	n/a	GCTACAGACCCTTTGCTCTG	GCTCATCTGTGTCGCTGTCC	first WNT3A CRISPR/Cas9n pair
WNT3A 2/2'	n/a	GATGGGCTGCGAGCCCAGGG	GTGTGCCAGCATCCCGGGCC	second WNT3A CRISPR/Cas9n pair
**gPCR screening primers spanning CRISPR-targeted regions**				
WNT3 gDNA span CR	246 WT	GGCAGAAGCGCAGTTGCTT	CCCGAGGTCAAAGGTAGCACA	
WNT3A gDNA span CR	247 WT	TTCATGGTGGAAGCTGCGT	TGGGCATGATCTCCACGTAG	
**RT-PCR primers for WNT3/3A knockout validation**				
WNT3 span ex2	528 WT / 286 mut.	CAGGGTCCTCGCTGGCTA	GCCTCGTTGTTGTGCTTGTT	PCR shown in Figure 4
WNT3A span ex2	491WT / 249 mut.	GCCCCACTCGGATACTTCTT	ACCATCCCACCAAACTCGAT	PCR shown in Figure 4
WNT3 in ex2	486 WT / mut. -	GGGCCAGCAGTACACATCTC	GCCTCGTTGTTGTGCTTGTT	PCR shown in Figure S4
WNT3A in ex2	404 WT / mut. -	CAGTATTCCTCCCTGGGC	ACCATCCCACCAAACTCGAT	PCR shown in Figure S4
**ChIP primers**				
WNT3 promoter	74	GGACGGAGCCGAGTGTCATT	ATGCAAAGGCAGCAGGAGGT	
WNT3A promoter	76	CACACCACGGATGAGTCTGGA	ACCGCGTTGGAATTGAGGAA	
OTX2	106	TCTGCCTTTGTCTTGGGATGC	GCCCATTCTTCTGCCAATTCA	irrelevant locus for qPCR normalization
