# Supplementary material for: Cardiogenic programming of human pluripotent stem cells by dose-controlled activation of EOMES
Source: Nat Commun. 2018 Jan 30;9:440. doi: 10.1038/s41467-017-02812-6 (PMC5789885; doi:10.1038/s41467-017-02812-6)
Supplement: Supplementary file 3 — Description of Additional Supplementary Files [file 41467_2017_2812_MOESM3_ESM.docx]

**Description of Additional Supplementary Files**

File name: Supplementary Data 1

Description: Non-cardiac mesoderm and endoderm-permissive differentiation of EOMES KO hESCs (day 12, filtered microarray data underlying Fig. 1d). Undifferentiated d 0 samples as well as differentiated WT data are shown for comparison. Filtering criteria: [> 10-fold intensity ratio vs. undifferentiated d 0 cells and signal intensity > 1000 a.u.] *or* [> 3-fold intensity ratio vs. differentiated WT cells *and* signal intensity > 100 a.u.]. Differentiation conditions were those indicated in Supplementary Fig. 1 (pooled samples, n = 1).

File name: Supplementary Data 2

Description: Cardiac induction time-courses (microarray data, n = 3 pooled samples from independent experiments per sample type). The data set comprises wild-type and EOMES knockout hESCs subjected to the standard protocol, and EOMES^KO/TET-ON^ cells differentiated via the TET-ON protocol. Data shown are normalised signal intensities and expression ratios against the standard condition (with conditional formatting).

File name: Supplementary Data 3

Description: Short-term signaling factor stimulation experiment (microarray data). Gene expression ratios and p values are colour-coded based on numerical thresholds. Statistics are based on bead s.d..

File name: Supplementary Movie 1

Description: EOMES TET-ON hESCs differentiated in a 24-well format via the DOX protocol (day 14).

File name: Supplementary Movie 2

Description: WT^E.TET-ON^ hESCs differentiated in 6-well format using the TET-ON protocol applied to a routine hESC culture (day 11).

File name: Supplementary Movie 3

Description: Replated pCMs derived from WT^E.TET-ON^ hiPSCs (day 13)
